# Developmental biology and potential use of *Alboglossiphonia lata* (Annelida: Hirudinea) as an “Evo-Devo” model organism

**DOI:** 10.1186/s12983-017-0240-y

**Published:** 2017-12-28

**Authors:** Brenda Irene Medina Jiménez, Hee-Jin Kwak, Jong-Seok Park, Jung-Woong Kim, Sung-Jin Cho

**Affiliations:** 1 School of Biological Sciences, College of Natural Sciences, Chungbuk National University, Cheongju, Chungbuk 28644 Republic of Korea; 2 Department of Life Science, College of Natural Sciences, Chung-Ang University, Seoul, 06974 Republic of Korea

**Keywords:** Embryonic development, Leech, Spiral cleavage, Mesodermal precursor, Ectodermal precursor, *Calsensin*, Neurogenesis, Evo-devo

## Abstract

**Background:**

The need for the adaptation of species of annelids as “Evo-Devo” model organisms of the superphylum Lophotrochozoa to refine the understanding of the phylogenetic relationships between bilaterian organisms, has promoted an increase in the studies dealing with embryonic development among related species such as leeches from the Glossiphoniidae family. The present study aims to describe the embryogenesis of *Alboglossiphonia lata* (Oka, 1910), a freshwater glossiphoniid leech, chiefly distributed in East Asia, and validate standard molecular biology techniques to support the use of this species as an additional model for “Evo-Devo” studies.

**Results:**

*A. lata* undergoes direct development, and follows the highly conserved clitellate annelid mode of spiral cleavage development; the duration from the egg laying to the juvenile stage is ~7.5 days, and it is iteroparous, indicating that it feeds and deposits eggs again after the first round of brooding, as described in several other glossiphoniid leech species studied to date. The embryos hatch only after complete organ development and proboscis retraction, which has not yet been observed in other glossiphoniid genera. The phylogenetic position of *A. lata* within the Glossiphoniidae family has been confirmed using cytochrome c oxidase subunit 1 (CO1) sequencing. Lineage tracer injections confirmed the fates of the presumptive meso- and ectodermal precursors, and immunostaining showed the formation of the ventral nerve system during later stages of development. Further, the spatiotemporal expression of an EF-hand calcium-binding protein Calsensin ortholog was characterized, which showed a specific pattern in both the ventral and peripheral nervous systems during the later stages.

**Conclusions:**

Our description of the embryonic development of *A. lata* under laboratory conditions provides new data for further comparative studies with other leech and lophotrochozoa model organisms. Moreover, it offers a basis for the establishment of this species as a model for future “Evo-Devo” studies.

**Electronic supplementary material:**

The online version of this article (10.1186/s12983-017-0240-y) contains supplementary material, which is available to authorized users.

## Background

The study of new non-model organisms such as annelids has gained more attention in recent years. Of the three bilaterian clades, namely, Deuterostomia, Ecdysozoa, and Lophotrochozoa [1, 2], the latter remains the least represented clade because of the preference for classic, genetic model organisms. This has led to gaps in understanding the evolutionary history of the bilaterians [3]. Developmental studies reveal a mixture of conserved and derived features, which are interpreted in light of the underlying phylogenetic relationships that are established independently by molecular phylogenetic analysis. Understanding of the actual mechanisms that shape development and evolution requires detailed knowledge of the cellular processes occurring during embryogenesis, a more highly resolved phylogenetic tree for annelids and their allies, and the inclusion of more species in comparative studies [4]. Earthworms and leeches have been studied to address this concern. This allows the establishment of new model organisms for the members of Lophotrochozoa such as *Helobdella austinensis* (Kutschera et al. 2013) [5], which provides a reference for studying satellite species [6].

*Alboglossiphonia lata* (Oka, 1910) [7] belongs to the Glossiphoniidae family, which is among the more species-rich leech families in terms of described numbers of species [8]. Worldwide, the presence of *A. lata* is primarily recorded in East Asia, including China, Japan, Taiwan, and South Korea, as well as in Hawaii [9, 10]. Glossiphoniidae leeches are characterized for having dorso-ventrally flattened and dorsally convex bodies, and for bearing a proboscis. These leeches usually feed on the blood of turtle or amphibians in clean, non-organic polluted streams, irrigation ditches, and open sewers. However, some glossiphoniids, like those belonging to the genera *Helobdella* (Blanchard, 1896) and *Glossiphonia* (Johnson, 1816), feed on the hemolymph of aquatic oligochaetes and snails [11]. Regarding parental care, all known Glossiphoniidae have evolved the habit of brooding the eggs and juveniles [12].

Studies on the embryonic development of the East Asian freshwater leech, *A. lata* have not been conducted yet. The present study aims to describe the embryonic development of the glossiphoniid *A. lata* under laboratory conditions. In addition, lineage tracer injection, immunostaining and gene expression experiments were performed to support the use of this species as an “Evo-Devo” model organisms in the future.

## Results

### Phylogenetic analysis

**Fig. 1:**
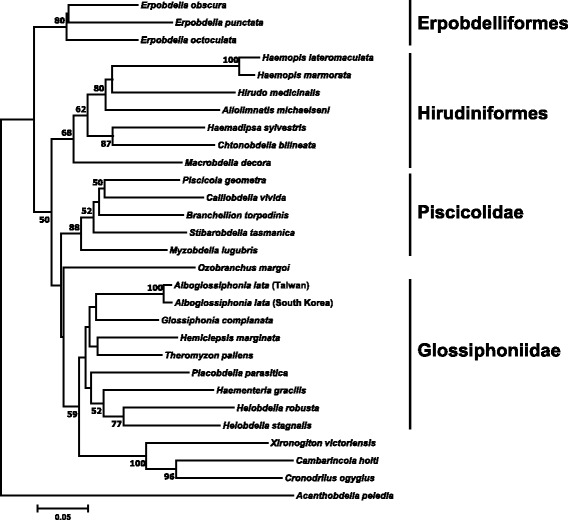
Phylogenetic position of *Alboglossiphonia lata* using cytochrome c oxidase CO1 sequencing. The evolutionary history was inferred using the Neighbor-Joining method [41]. The percentage of replicate trees in which the associated taxa clustered together in the bootstrap test (1000 replicates) are shown next to the branches [42]. The tree is drawn to scale, with branch lengths in the same units as those of the evolutionary distances used to infer the phylogenetic tree. The evolutionary distances were computed using the Maximum Composite Likelihood method [45] and are in the units of the number of base substitutions per site. The analysis involved 29 nucleotide sequences. All positions containing gaps and missing data were eliminated. There were a total of 475 positions in the final dataset. Evolutionary analyses were conducted in MEGA7 [44]

Phylogenetic analysis using the Neighbor-Joining method was conducted to determine the evolutionary history of *A. lata*, resulting in a consensus tree (Fig. 1), in which *Acanthobdella peledia* was used as outgroup. This tree overall corroborated the relationships traditionally suggested for leeches by morphology [13]. And, both *A. lata* CO1 sequences from Taiwan and South Korea were clustered together alongside the type species of *Glossiphonia*, *Glossiphonia complanata* (Linnaeus, 1758) within the Glossiphoniidae family.

### Embryonic development

The nomenclature used in the present study to describe the developmental stages of *A. lata* embryos follows the standard embryonic staging system devised for glossiphoniid leeches [3, 14–16]. A table listing the brooding period for several leeches species [3, 12, 17–23] including *A. lata* is provided (Table 1). Like all leeches, *A. lata* presents direct development. Embryos are ~0.5 mm in diameter and protected by a transparent cocoon on the ventral side of the parent leech. Cocoons contained from 15 to 116 embryos, with a mean of 47 ± 23 (*n* = 100). Direct observation showed that the number of embryos inside a cocoon increases with the size of the adult. *A. lata* embryos also present a light yellowish, at times greenish, coloration. Hypodermic insemination has been observed for many glossiphoniid species. During copulation, the spermatophores are usually released through an ejaculatory duct in the clitellar region of the concopulant or implanted anywhere in the posterior part of the leech body [24]. The spermatozoa are then released from the spermatophore and reach the ovaries through the vector tissue [19]. No spermatophores were observed to be attached to adult leeches in our laboratory population during this study. Prior to egg fertilization, adult specimens group together, with the dorsal side of one leech being covered by the ventral surface of the other.

**Table 1:** Comparison between brooding periods of several Glossiphoniidae species

Species	Brooding period
*Alboglossiphonia lata*	7.5 days
*Alboglossiphonia hyalina* [17]	11–12 days
*Alboglossiphonia polypompholyx* [18]	15 days
*Batracobdella algira* [19]	20 days
*Glossiphonia complanata* [12]	3–4 weeks
*Oligobdella biannulata* [20]	10–20 days
*Theromyzon tessulatum* [21]	2–3 weeks
*Helobdella striata* [22]	6–12 days
*Helobdella austinensis* [3]	10 days
*Helobdella robusta* [3]	13 days
*Helobdella stagnalis* [23]	24 days

### Cleavage (stages 1 to 6)

**Fig. 2:**
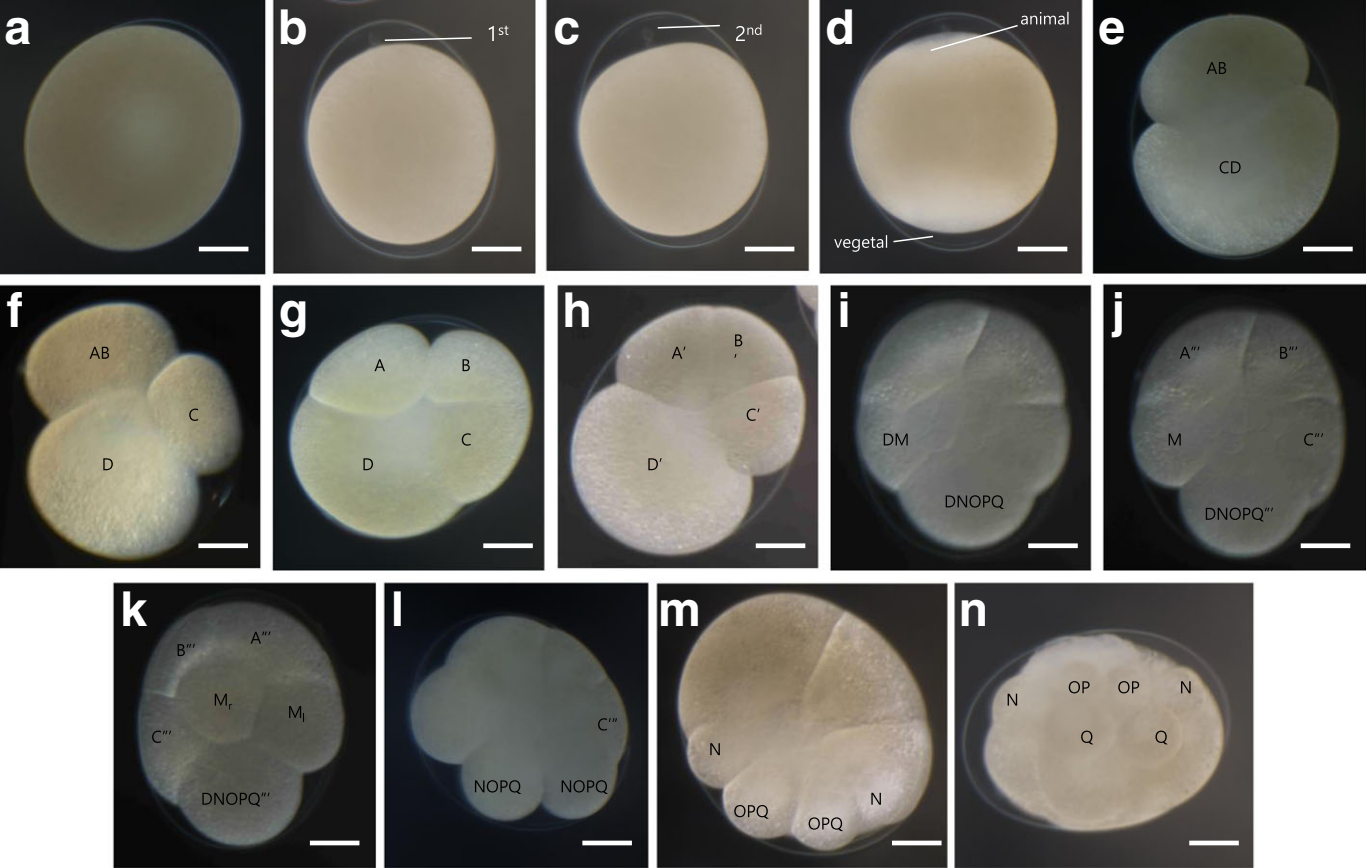
Cleavage of *A. lata* embryo. **a** Early stage 1 after zygote deposition. **b** First polar body formation. **c** Second polar body formation. **d** Teloplasm formation during late stage 1. **e** Stage 2 where the first asymmetric cleavage (AB + CD) occurs. **f** Early stage 3, in which the second asymmetric cleavage (C + D) takes place. **g** Late stage 3 showing the third asymmetric cleavage (A + B) with teloplasm being segregated to the D macromere. **h** Stage 4a, in which the first micromere quartet is formed. **i** Stage 4b, defined by the obliquely horizontal cleavage of macromere D’ which forms meso and ectodermal precursors DM and DNOPQ. **j** Animal view of stage 4c showing the right M teloblast, originated from DM proteloblast, and DNOPQ’ cell. **k** Vegetal view of stage 4c showing the left M teloblast. **l** Division of cell DNOPQ"' into left and right NOPQ proteloblasts during Stage 5. **m** NOPQ" cells form N teloblasts and OPQ proteloblasts in Stage 6a. **n** Stage 6b, marked by the division of OPQ” into OP proteloblasts and Q teloblasts. All images in animal view unless indicated otherwise. Scale bar in all images 100 μm

**Fig. 3:**
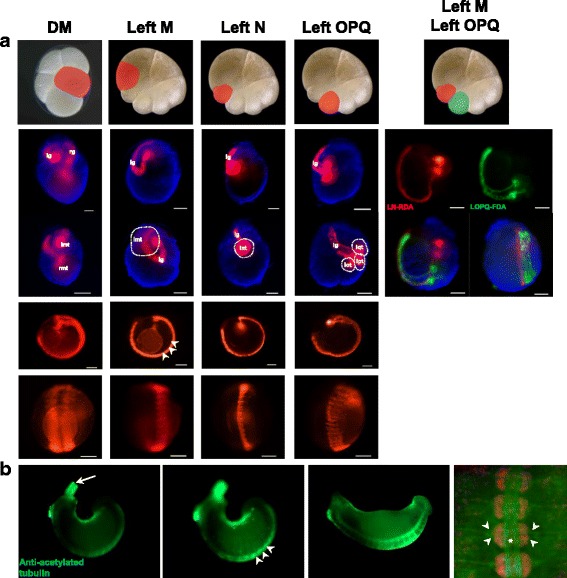
Fluorescent lineage tracer injection and Anti-acetylated tubulin stained *A. lata* embryo. To show the broad lineage of DM cell, we injected the RDA in DM cell at stage 4b. DM cells divided into left and right mesodermal precursor cells. DM cells were divided to M_l_ and M_r_ at stage4c. After then, M lineage precursor cells organize the mesodermal lineage tissue and have overall function of mesoderm lineage. **a** To confirm the exact function of M lineage precursor cell in *A. lata* embryos, we injected left M cells at stage 6a. M cells have a function to develop mesodermal lineage tissue (muscle fiber, mesodermal prickle cell). N cells have a potential to develop neuronal tissue, presumptive neuronal ganglion. OPQ cells have function of neuro-ectodermal lineage, exterior region of N lineage. To confirm the separative lineage of N and OPQ, we injected double lineage tracer at stage 6a. N and OPQ cells have individual lineage in developing *A. lata* embryos; lg: left germinal band; rg: right germinal band; rmt: right M teloblast; lmt: left M teloblast; lnt: left N teloblast; lot: left O teloblast; lpt: left P teloblast; lqt: left Q teloblast. Scale bar in all images 100 μm. **b** To show *A. lata* as the experimental model, we stained late stage embryos using Anti-acetylated tubulin. At stage 10, Anti-acetylated tubulin stained nerve fiber in the everted proboscis. Out focused embryos show that Anti-acetylated tubulin has a role in ventral ganglion and peripheral nerve fiber. At stage 11, Anti-acetylated tubulin is expressed in overall part of embryo. We pseudo-colorized the DAPI stained ventral ganglion at stage 11 for optimizing the peripheral nerve and central nerve-ganglion. Scale bar in all images 100 μm

After fertilization, meiosis is arrested at metaphase I until zygotes are deposited sequentially, leading to a slight asynchrony among the embryos of a single clutch. Before egg laying occurs, the parent leech squeezes a membranous sac, or cocoon, which encloses the incoming eggs. Consecutive extrusion of the two polar bodies, immediately followed by initiation of teloplasm formation [25] marks the completion of Stage 1 (Fig. 2a-d). Cleavage occurs until the formation of teloblasts, the ten embryonic stem cells that give rise to the segmental mesoderm and ectoderm. In *A. lata* embryos, two unequal cleavages segregate the teloplasm to the macromere D, constituting Stages 2 and 3 (Fig. 2e-g). An animal pole quartet of micromeres (a’ - d’) is then generated by the first, highly unequal, dextro-rotatory spiral cleavage; this constitutes Stage 4a (Fig. 2h). The formation of cells DNOPQ and DM is attributed to the obliquely horizontal cleavage of macromere D’ (Fig. 2i). The end of Stage 4b is marked by each of the three A, B, and C quadrants forming three micromeres (a’ - a’“, b’ - b’“, c’ - c’“) following which macromeres A’“, B’“, and C′“stop dividing, and then contribute to midgut endoderm formation during the later stages. Injection of dextran,tetramethylrhodamine (RDA) in DM" cells at Stage 4b confirmed the division of cell DM" into left and right M teloblasts, constituting the beginning of Stage 4c (Fig. 2j-k). The right M teloblast is located near the center of the vegetal pole, whereas the left M teloblast is visible from the animal pole. Stage 5 is characterized by the formation of more micromeres and the division of the cell DNOPQ”‘into left and right NOPQ cells (Fig. 2l). Subsequent division of the ectodermal precursors generates OP proteloblasts and Q teloblasts, marking Stage 6a (Fig. 2m). The differences in the lineage of the N teloblast and OPQ proteloblast were confirmed by double lineage tracer injection at this stage (Fig. 3a). By the end of Stage 6 (Fig. 2n), *A. lata* embryo comprises more than 38 cells as a result of further division of macromeres, which later contribute to the non-segmental, dorsal anterior ganglion of the nervous system. Through RDA injections on N and OPQ cells, it was possible to verify that during later stages of development, N cells differentiate into neuronal tissue and presumptive neuronal ganglions. On the other hand, OPQ cells differentiate into neuro-ectodermal-derived cells, which constitutes the exterior region of N lineage (Fig. 3a).

### Germinal band formation (stages 7 to 8)

**Fig. 4:**
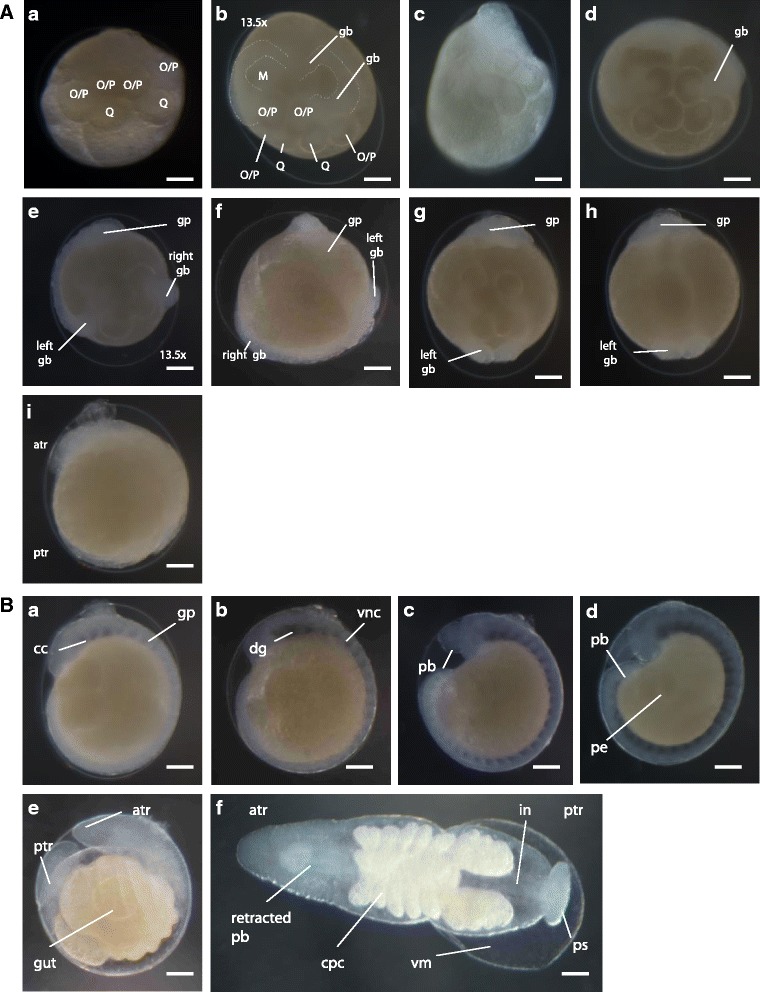
**A** Germinal bands and germinal plate formation of *A. lata* embryo. (**a**) Early stage 7 (**b**) late stage 7. (**c**) Lateral view. (**d**) Early stage 8. (**e**) Middle stage 8 dorsal view. (**f**) Middle stage 8 ventral view. (**g**) Late stage 8 dorsal view. (**h**) Late stage 8 ventral view. (**i**) Late stage 8 lateral view. Scale bar in all images 100 μm. **B** Organogenesis and hatching of *A. lata* embryo. (**a**) Lateral view of early stage 9 embryo, indicating the coelomic cavities (cc) arising within the mesoderm in anterior to posterior progression after the completion of germinal plate (gp) formation. (**b**) Lateral view of middle stage 9 embryo, showing the formation of the ventral nerve cord (vnc) and dorsal ganglion (dg). (**c**) Lateral view of early stage 10 embryo, showing the everted proboscis (pb). (**d**) Lateral view of late stage 10 embryo in which the everted proboscis is more notorious and the provisional epithelium is being displaced towards the dorsal midline. (**e**) Lateral view of early stage 11 embryo showing proboscis retraction and gut formation. (**f**) Dorsal view of middle stage 11 embryo showing the crop ceca (cpc), intestine (in) and posterior sucker (ps) formed, also showing detachment of the vitelline membrane (vm). Anterior is up in all views unless indicated otherwise. Scale bar in all images 100 μm

Stage 7 begins with the equal division of OP proteloblasts, forming pairs of O/P teloblasts (Fig. 4Aa). Multiple unequal divisions of each teloblast give rise to blast cells, which will form bandlets that later constitute the germinal bands (Fig. 4Ab-c). These then come in contact with each other through their distal ends at the region where the future head of embryo will develop [3]. The germinal bands start forming during the later part of Stage 7. The diameter of the formed O/P cells decreases as they start producing blast cells, observed by light microscopy as more transparent bands growing from each cell. RDA injection to M cells confirms that the pair of mesoteloblasts (M) gives rise to the mesodermal-derived tissue, namely, muscle fiber and prickle cell (Fig. 3a). By the time Stage 8 begins, the ectodermal and mesodermal bandlets reach the surface of the embryo; the ectodermal bandlets on each side seem to crawl distally along the ipsilateral m bandlet to start forming the germinal bands, culminating in the formation of the germinal plate (Fig. 4Ae-i). Epiboly constitutes the main event in Stage 8. Additionally, during the later part of Stage 8, the embryonic attachment organ is formed at the anterior end of the germinal plate and is the first part of the embryo to emerge from the vitelline membrane (Fig. 4Aj). This organ can be pushed into the ventral surface of the parent leech, allowing the developing embryo to be carried by the parent adult until hatching, which happens by the time the suckers are strong enough to clamp onto the ventral surface of the parent [26].

### Organogenesis and hatching (stages 9 to 11)

**Fig. 5:**
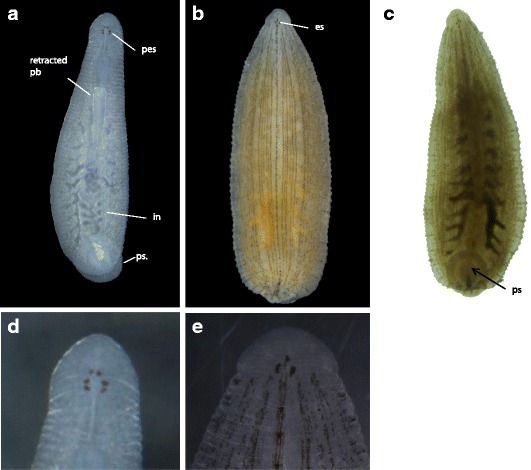
Post-embryonic developmental stage of *A. lata* embryo. **a** Dorsal view of a juvenile showing the retracted proboscis (pb), reddish pigmented eye spots (pes), intestine (in) and posterior sucker. **b** Dorsal view of an adult indicating the black eye spots (es). **c** Ventral view of an adult indicating the posterior sucker. Anterior is up in all views. **d** Dorsal view of head region showing reddish pigmented eye spots in juvenile. **e** Dorsal view of head region showing blackish eye spots in adult. Scale bar 100 μm (**a**-**c**); Scale bar 50 μm (**d**, **e**)

Stage 9 starts with the completion of the germinal plate formation. It is distinguished by the appearance of bilateral pairs of coelomic cavities within the mesoderm, progressing from the anterior to the posterior end, and it culminates after the ventral cord is visible and connects anteriorly to the dorsal ganglion by the circumesophageal connective nerves (Fig. 4Ba-b). The beginning of Stage 10 is characterized by the formation of the posterior-most coelomic cell layer, whose proliferation lead to the lateral and dorsal expansion of the edges of the germinal plate; this, gradually displaces the provisional epithelium formed during Stage 8, toward the dorsal midline. The proboscis differentiates into an everted position (Fig. 4Bc-d). Immunostaining using anti-acetylated tubulin allowed the visualization of the formation of the ventral ganglion and peripheral nerve fibers during this stage (Fig. 3b). Stage 11 starts when the lateral edges of the germinal plate have met all along the dorsal midline. During Stage 11, the proboscis invaginates. Development of the ventral ganglion and peripheral nerves is completed, and the crop ceca, intestine, posterior sucker, and pigmented eye spots are formed (Fig. 4Be). Only after *A. lata* develops eye spots and retracts its tubular proboscis inside the mouth orifice, it hatches from the vitelline membrane (Fig. 4Bf). *A. lata* develops three pairs of eye spots, with the anterior-most pair being close together. The newly formed eye spots are colored red and turn dark brown soon after the embryo hatches (Fig. 5d-e).

### Post-embryonic stage and parental care

The beginning of juvenile stage is marked by the exhaustion of the yolk from within the crop [27]. Starting from mid Stage 11, *A. lata* grows distinctively flat (Fig. 5a-c). Body coloration of the growing adults is fawn to pale, slightly translucent with a body length of 10–22 mm [9]. Parental care in *A. lata* ends after the juveniles that have exhausted their yolk start leaving the adult. After brooding is finished, the parent adults feed on snails and reproduce at least two times more before dying.

### Calsensin expression patterns

**Fig. 6:**
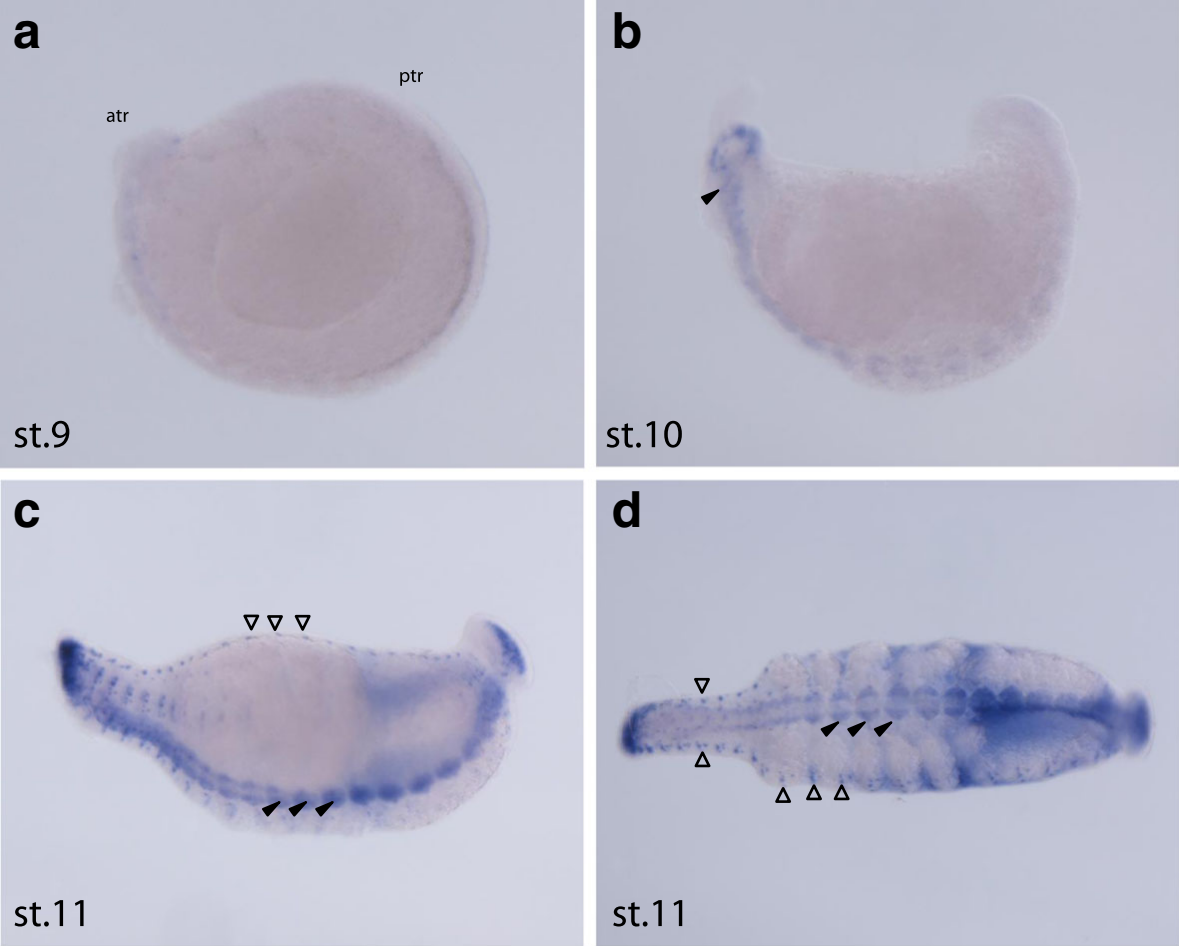
Expression pattern of *Ala-calsensin* during late embryonic development of A. lata (from stage 9 to 11). **a** No staining detected at stage 9 (lateral view); atr: anterior, ptr: posterior. **b** Arrow head indicating staining in the developing segmental ganglia at stage 10 (lateral view). **c** At stage 11, *Ala-calsensin* expression was detected in the segmental ganglia (black arrowheads) as well as peripheral neurons in the body wall (red arrowheads) (lateral view). **d** Ventral view of stage 11 embryo showing *Ala-Calsensin* expression in the segmental ganglia (black arrowheads) and peripheral neurons in the body wall (red arrowheads). Scale bar in all images 100 μm

Chemical in situ studies for *Ala-calsensin* expression were conducted during the later stages of *A. lata* development. No expression was detected during Stage 9 (Fig. 6a). During Stage 10, expression of mRNA transcripts was detected in the developing segmental ganglia (Fig. 6b). Then, during Stage 11, *Ala-calsensin* was expressed in the segmental ganglia and the peripheral neurons in the body wall (Fig. 6c-d). In addition, a phylogenetic tree was constructed that clusters *Ala-calsensin* and a *Helobdella robusta calsensin* ortholog together to form a monophyletic group alongside *Hma-calsensin* within Hirudinea (BP > 50%) (Additional file 1).

## Discussion

DNA markers from mitochondrial genomes, like cytochrome C oxidase subunit 1 (CO1), are widely used for estimating phylogenetic relationships among closely allied taxa [28]. In the present study, the consensus tree generated by Neighbor-Joining method has confirmed the phylogenetic relationships within the Glossiphoniidae family established by Siddall and collaborators [29], in which *A. lata* species cluster alongside *G. complanata* in a monophyletic group that shares the common trail of presenting three pairs of eyespots. Our results are supported by a pairwise-distance matrix (Additional file 2) [30].

The developmental process observed in *A. lata* embryos is overall similar to that observed in the model organisms *H. austinensis* and other studied glossiphoniidae leech species. Aggregated groups of mature individuals were similar to those observed in *A. hyalina* [17], suggesting that hypodermic insemination [19] occurs in *A. lata*, although this was not directly observed. Nagao (1958) observed that the cocoon of *A.lata* is secreted from the clitellar glands surrounding the female gonopore [31], which is also the case for at least one *Helobdella* species [22]. Like all glossiphoniids, *A. lata* develops an embryonic attachment organ [22, 31]. This organ, formed at the later part of stage 8, appears to be more prominent than that of other glossiphoniids such as *Helobdella*. Although microinjection experiments in *A.lata* embryos were ultimately successful, their vitelline membrane proved to be more difficult to puncture than that of *H. austinensis* embryos. To overcome this issue, better injection skills and the making of more resistant and sharper needles were required. At the same time, it was observed that injected *A. lata* embryos showed more resistance to bacterial infection in comparison to injected *Helobdella* embryos. The vitelline membrane of *A. lata* being thicker than that of *H. austinensis* could have helped decreasing the risk of infection of *A. lata* embryos after injection. Hatching of *A. lata* appears to be delayed relative to other glossiphoniid species outside of *Alboglossiphonia* [17]. The eye spots, midgut diverticula and at least the posterior sucker are well differentiated prior to hatching (Fig. 43Be, Bf). Allowing the embryo to fully develop inside the vitelline membrane could potentially increase the chances of survival in case the embryos were forcefully or accidentally detached from the ventral surface of the parent adult before hatching, in comparison to those species that hatch earlier. We speculate that this delay could have been an evolutionary advantage for the *Alboglossiphonia* genus. Regarding reproduction strategy, *A. lata* leeches have shown to be iteroparous, with at least two reproductive cycles before dying. This strategy is known in other glossiphoniid genera [32]. Interestingly, several studied *Alboglossiphonia* species [17, 18, 33, 34] and at least one *Helobdella* [22] and *Batracobdella* [19] species are, in contrast, semelparous. It is important to note that our observations occurred under experimental conditions, and therefore, it is recommended to elaborate a methodology for future confirmatory studies in *A. lata* specimens in situ.

The present study, for detailed comparative purposes with current leech model organisms species (Additional file 3, Table 1), followed the stage division known for *Helobdella* [3], in which eleven stages are established. However, in similar embryogenesis studies conducted for other leech species, this stage division varied [17, 34]. 

Calsensin is a EF-hand calcium-binding protein that was first found in the leech *Haemopis marmorata*, and is thought to mediate calcium-dependant signal transduction events in growth cones and axones of the developing nervous system [35]. The spatial expression of an EF-hand calcium-binding protein Calsensin ortholog in *A. lata* (*Ala-calsensin*) has been characterized, appearing to be expressed in the segmental ganglia and peripheral neurons in the body wall during organogenesis  (10 and 11). Considering that Calsensin expression has been detected in central and peripheral nerves of other hirudinid species [36, 37], our results give further support for a potential physiological role of Calsensin in the formation and maintenance of nerve pathways in leech species.

Successful injection of lineage tracing, visualization of neurogenesis during later stages by immunostaining using anti-acetyl tubulin antibody, and spatial expression pattern-based characterization of Calsensin by chemical in situ hybridization support the use of *A. lata* as a model organisms for “Evo-Devo” studies.

## Conclusions

Description of the embryonic development of *A. lata* in vitro provides new data for further comparative studies involving other leech species. In addition, successful use of molecular biological techniques, such as microinjection of embryos for lineage tracing, in situ hybridization for spatial gene expression, and immunostaining for neurogenesis offers a basis for the development of this leech species as an “Evo-Devo” model organisms in the future.

## Methods

### Leech breeding

Adult specimens, collected in nearby ponds and purchased online from Yeosu Aquarium, were bred in the Laboratory of Cellular and Developmental Biology (LCDB) of the Department of Biology of Chungbuk National University, Republic of Korea. Following the Protocol for Handling of *Helobdella* (Leech) embryos [38], *Alboglossiphonia lata* adult specimens (body length at rest: 10 – 18 mm) were deposited in Petri dishes with lid containing Working Solution. The specimens were cleaned once a day by changing the culture medium and scrubbed manually to get rid of any residual waste, and kept in an incubator at 22 °C. Their diet consisted of red snails purchased online, which were bred in fish bowls with Working Solution at room temperature.

Gravid adults were carefully manipulated with blunt forceps in order to remove the cocoons adhered to their ventral body wall. With the help of a sterilized pipette, each cocoon was transported to a separate smaller petri dish containing HTR medium for further examination and culture.

Using sterilized insect pins. Embryos were detached from their respective cocoons and observed through a Leica ZOOM 2000 stereomicroscope to identify their current developmental stage. Expecting a similarity with the timing of developmental stages in *Helobdella* species, *A. lata* embryos were checked every half an hour since deposition until it reached stage 4, then they were checked at least twice a day for the following stages. Embryos were imaged at each developmental stage using a Nikon SMZ18 stereomicroscope.

In order to elaborate a timeline for *A. lata* developmental stages that can be compared to the existing ones for *Helobdella robusta* and *Helobdella austinensis*, each one of the stages was indicated in terms of the number of hours after zygote deposition (AZD).

The nomenclature used in the present study to describe the developmental stages of *A. lata* embryos follows the standard embryonic staging system devised for glossiphoniid leeches [3, 14–16].

### CO1 gene cloning and sequencing

Total RNA from *Alboglossiphonia lata* embryos was isolated using TRIzol (Ambion). Then mRNA from RNA using Oligo (dT) primer (Promega) was selected, and reverse transcription into cDNA (SuperScript II First-Strand Synthesis System for RT-PCR, Invitrogen) was conducted. CO1 protein coding homologous sequences were searched on sequenced RNA database available in our laboratory of Cellular and Developmental Biology (LCDB). Specific primers (CO1_Contig2 Forward: 5′ GCAGTGAAATATGCTCGGGT 3′, CO1_Contig2 Reverse: 5′ GAGTTAGCACAACCAGGCTCA 3′) were designed in order to amplify CO1 from *A. lata* cDNA. We followed the TaKaRa protocol for PCR according to standard procedure.

### Phylogenetic analysis

In order to acquire CO1 sequences, we cited the Leech gene sequencing articles available [29, 39] and used the accession numbers from said articles, with the exception of the *Hirudo medicinalis* CO1 sequence, because it had many gaps and did not qualify. Instead, we used the nucleotide sequence under the accession number AY786458 from NCBI that does not present gaps and is shorter than the previously referenced sequence. We searched additional accession numbers not present in these articles in NCBI. The sequences were aligned and trimmed using biological sequence editor BioEdit (http://www.mbio.ncsu.edu/BioEdit/bioedit.html). Aligned sequences were analyzed in MEGA7.

### Calsensin gene identification, gene cloning, probe synthesis, whole-mount in situ hybridization and nuclear staining

Total RNA was isolated from *Alboglossiphonia* species (*lata*) embryos of different developmental stages using TRIzol (Ambion). We selected mRNA from RNA using Oligo (dT) primer (Promega), and then conducted reverse transcription into cDNA (SuperScript II First Synthesis System for RT-PCR, Invitrogen). To demonstrate the feasibility of molecular approaches to *A. lata*, we isolated an EF-hand motif Calsensin gene, conducted alignment using alignment tool ClustalW, and phylogenetic analysis using tool MEGA7. After confirmation, the investigated leech gene was isolated by means of PCR, using gene-specific primers (*Ala-calsensin* Forward: 5′ GCCAAACGTTACCGAACCTCG 3′; *Ala-calsensin* Reverse: 5′ GAGAAGGTCCGCGTTGGCG 3′) based on sequenced RNA database available in our laboratory of Cellular and Developmental Biology (LCDB). The amplified fragments were cloned into pGEM T vector (Promega). Dioxigenin-labelled RNA probe were synthesized from the cloned fragments. Then in situ Hybridization (ISH) was performed as previously described [40]. Pre-hybridization was performed at 64.7 °C for one day in hybridization buffer (50% Formamide, 5× SSC, 1× Denhardt’s Solution, 0.1% CHAPS, 100 mg/ml Heparin, 0.1% Tween20, 100 mg/ml tRNA). The prehybridized buffer was replaced with fresh hybridization buffer containing 2 ng/ml of the corresponding probe and embryos were hybridized at 64.7 °C for 2 days. Washed embryos were incubated at room temperature for 2 h in 1% Blocking Regent dissolved in PBT (1× PBS plus 0.1% Tween20) then incubated at 4 °C for 16 h with 1/1000 Anti-DIG/AP in 1% Blocking Reagent. After incubation, the color reaction was carried out using nitro blue tetrazolium chloride/ 5-bromo-4-chloro-3-indoyl-phosphate (Roche) by standard procedures. Stained embryos were dehydrated in ethanol, mounted in plastic embedding solution (PolyBed, Roche), and examined by bright field microscopy on a Nikon SMZ18 stereomicroscope.

### Lineage tracing

Main cells were injected using dextran,tetramethylrhodamine (Molecular probes, D1817) for colorizing red. To visualize the different lineage, we injected green color fluorescence dye, the dextran Alexa fluor 488 (Molecular probes, D22910) in left OPQ cell at stage 6a when cleaved NOPQ to N and OPQ. After injection, the embryo were incubated at 22 °C in antibiotic (Gibco, 15,240,062) treated *Helobdella triserialis* media. After reaching the desired stage, embryos were fixed and treated with DAPCO glycerol. We labeled the embryo’s bright field color in pseudo blue color to pinpoint the exact location of the embryo. Embryos were imaged by fluorescent microscopy on a Nikon SMZ18 Stereomicroscope.

### Immunostaining

After rehydrating the embryos (stage 9 to 11), they were pre-incubated in 5% mercaptoethanol and 1% Triton in 0.1 M Tris-HCl (pH 7.5) at 37 °C on shaking incubator (rpm60) for an hour. Following three washes with PBT, the embryos were incubated in Block solution (1:9 10X Roche Western Blocking Reagent in PBT) for two hours. Then, embryos were incubated with a monoclonal anti-acetylated-α-Tubulin antibody (Sigma, T-7451) in Blocking Solution (1:500) at 4 °C for 72 h. After three consecutive washes with PBT, embryos were incubated with a secondary antibody (Abcam, ab150113) in Blocking Solution (1:1000) at 4 °C for 48 h. Consequently, embryos were washed overnight with PBT and then dyed with DAPI in PBT (1:1000) at room temperature in the dark for overnight. After washing with PBT three more times, embryos were finally embedded in 30%, 50% 20 min and 87% glycerol and 2.5 mg/ml of DABCO in 1xPBS. Embryos were imaged by fluorescence microscopy on a Nikon SMZ18 Stereomicroscope and LEICA DM6 B.

## Additional files

Additional file 1: Phylogenetic tree of *Ala-Calsensin*. The evolutionary history was inferred using the Neighbor-Joining method [41]. The percentage of replicate trees in which the associated taxa clustered together in the bootstrap test (1000 replicates) are shown next to the branches [42]. The tree is drawn to scale, with branch lengths in the same units as those of the evolutionary distances used to infer the phylogenetic tree. The evolutionary distances were computed using the Poisson correction method [43] and are in the units of the number of amino acid substitutions per site. The analysis involved 8 amino acid sequences for *Alboglossiphonia lata* (*Ala-calsensin*), *Helobdella robusta* (*Hro-calsensin* protein id: 185,720), *Haemopsis marmorata* (Hma-calsensin protein id: AAC46630.1), *Xenopus laevis* (*Xla-plastin3* protein id: NP_001083581.1), *Cricetulus griseus* (*Cgr-plastin3* protein id: ERE65879), *Anoplophora glabripennis* (*Agl-calbindin* protein id: JAB67778), *Drosophila melanogaster* (*Dme-calbindin32* protein id: AAA15214.1), *Hydra vulgaris* (*Hvu-calbindin* protein id: CDG71500). All ambiguous positions were removed from each sequence pair. There were a total of 318 positions in the final dataset. Evolutionary analyses were conducted in MEGA7 [44]. (PPTX 62 kb)

Additional file 2: Estimates of Evolutionary Divergence between Hirudinea sequences. Analyses were conducted using a Pairwise distance matrix [43]. The rate variation among sites was modeled with a gamma distribution (shape parameter = 1). The analysis involved 29 nucleotide sequences. All positions containing gaps and missing data were eliminated. There were a total of 475 positions in the final dataset. Evolutionary analyses were conducted in MEGA7 [44]. (XLSX 13 kb)

Additional file 3: Comparative Timeline of three glossiphoniidae leech species (*Alboglossiphonia lata*, *Helobdella robusta* and *Helobdella austinensis*) development from egg deposition (stage 1 at 0 h after zygote deposition (AZD)) until yolk-depleted juvenile. The embryonic development of *A. lata* is shorter than the one of both *H. robusta* and *H. austinensis*, with an approximate duration of seven and a half days (~180 h), compared to the approximated 9 and a half days (~229 h) for *H. robusta* and 13 days (~310 h) for *H. austinensis*. In *A. lata*, the vitelline membrane protecting the developing embryo is only broken during early stage 11 after the embryo has developed its eye spots and inverted its proboscis. In both *H. robusta* and *H. austinensis*, the vitelline membrane is broken by the maturing embryo somewhere between late stage 9 and early 10. (PPTX 81 kb)
