# Supplementary material for: Developmental biology and potential use of Alboglossiphonia lata (Annelida: Hirudinea) as an “Evo-Devo” model organism
Source: Front Zool. 2017 Dec 28;14:60. doi: 10.1186/s12983-017-0240-y (PMC5745604; doi:10.1186/s12983-017-0240-y)
Supplement: Supplementary file 1 — Phylogenetic tree of Ala-Calsensin. The evolutionary history was inferred using the Neighbor-Joining method [41]. The percentage of replicate trees in which the associated taxa clustered together in the bootstrap test (1000 replicates) are shown next to the branches [42]. The tree is drawn to scale, with branch lengths in the same units as those of the evolutionary distances used to infer the phylogenetic tree. The evolutionary distances were computed using the Poisson correction method [43] and are in the units of the number of amino acid substitutions per site. The analysis involved 8 amino acid sequences for Alboglossiphonia lata (Ala-calsensin), Helobdella robusta (Hro-calsensin protein id: 185,720), Haemopsis marmorata (Hma-calsensin protein id: AAC46630.1), Xenopus laevis (Xla-plastin3 protein id: NP_001083581.1), Cricetulus griseus (Cgr-plastin3 protein id: ERE65879), Anoplophora glabripennis (Agl-calbindin protein id: JAB67778), Drosophila melanogaster (Dme-calbindin32 protein id: AAA15214.1), Hydra vulgaris (Hvu-calbindin protein id: CDG71500). All ambiguous positions were removed from each sequence pair. There were a total of 318 positions in the final dataset. Evolutionary analyses were conducted in MEGA7 [44]. (PPTX 62 kb) [file 12983_2017_240_MOESM1_ESM.pptx]

## Slide 1
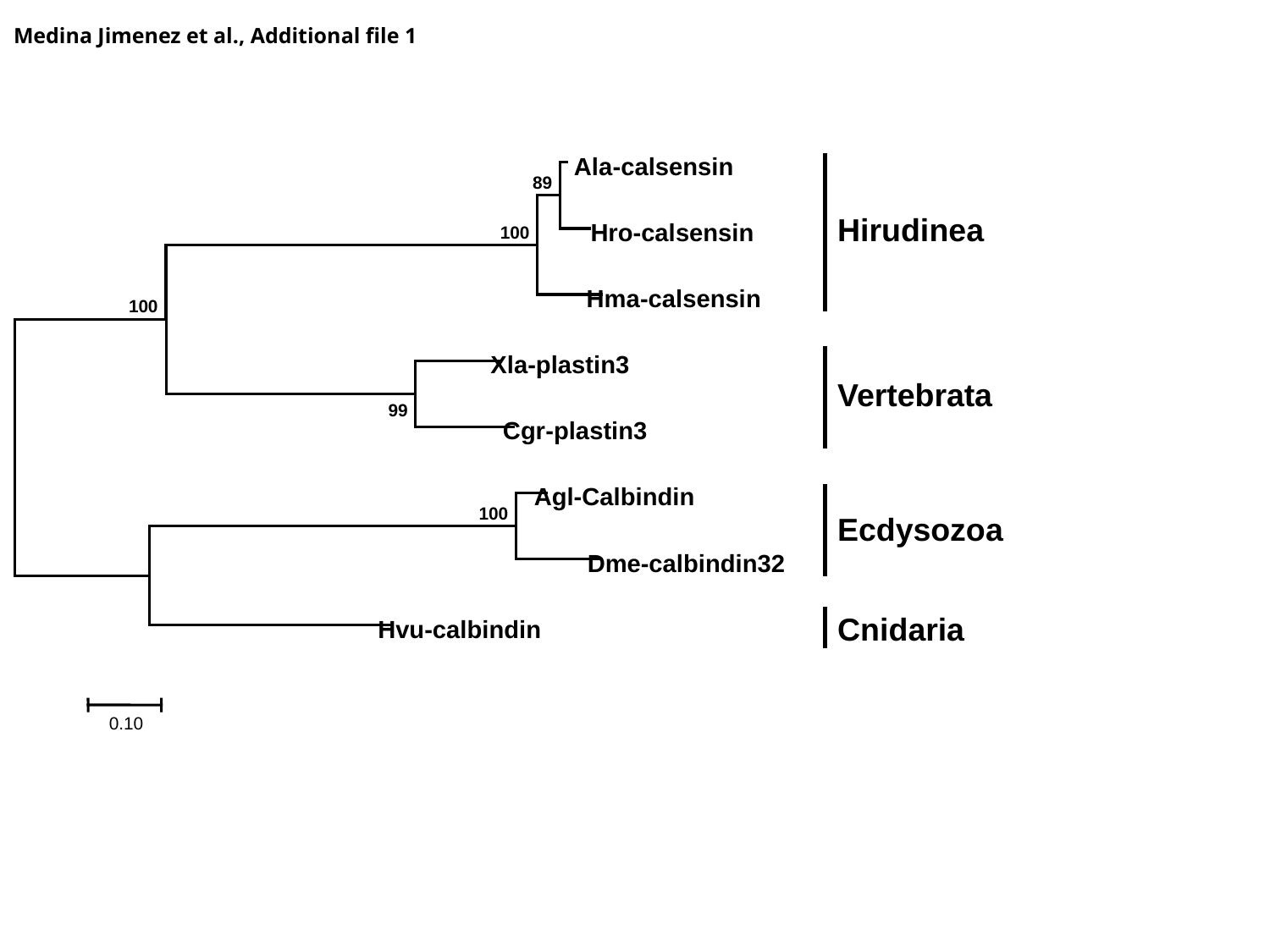

Medina Jimenez et al., Additional file 1
 Ala-calsensin
89
Hro-calsensin
100
 Hma-calsensin
100
 Xla-plastin3
99
 Cgr-plastin3
 Agl-Calbindin
100
 Dme-calbindin32
 Hvu-calbindin
0.10
Hirudinea
Vertebrata
Ecdysozoa
Cnidaria
