# Supplementary material for: Developmental biology and potential use of Alboglossiphonia lata (Annelida: Hirudinea) as an “Evo-Devo” model organism
Source: Front Zool. 2017 Dec 28;14:60. doi: 10.1186/s12983-017-0240-y (PMC5745604; doi:10.1186/s12983-017-0240-y)
Supplement: Supplementary file 3 — Comparative Timeline of three glossiphoniidae leech species (Alboglossiphonia lata, Helobdella robusta and Helobdella austinensis) development from egg deposition (stage 1 at 0 h after zygote deposition (AZD)) until yolk-depleted juvenile. The embryonic development of A. lata is shorter than the one of both H. robusta and H. austinensis, with an approximate duration of seven and a half days (~180 h), compared to the approximated 9 and a half days (~229 h) for H. robusta and 13 days (~310 h) for H. austinensis. In A. lata, the vitelline membrane protecting the developing embryo is only broken during early stage 11 after the embryo has developed its eye spots and inverted its proboscis. In both H. robusta and H. austinensis, the vitelline membrane is broken by the maturing embryo somewhere between late stage 9 and early 10. (PPTX 81 kb) [file 12983_2017_240_MOESM3_ESM.pptx]

## Slide 1
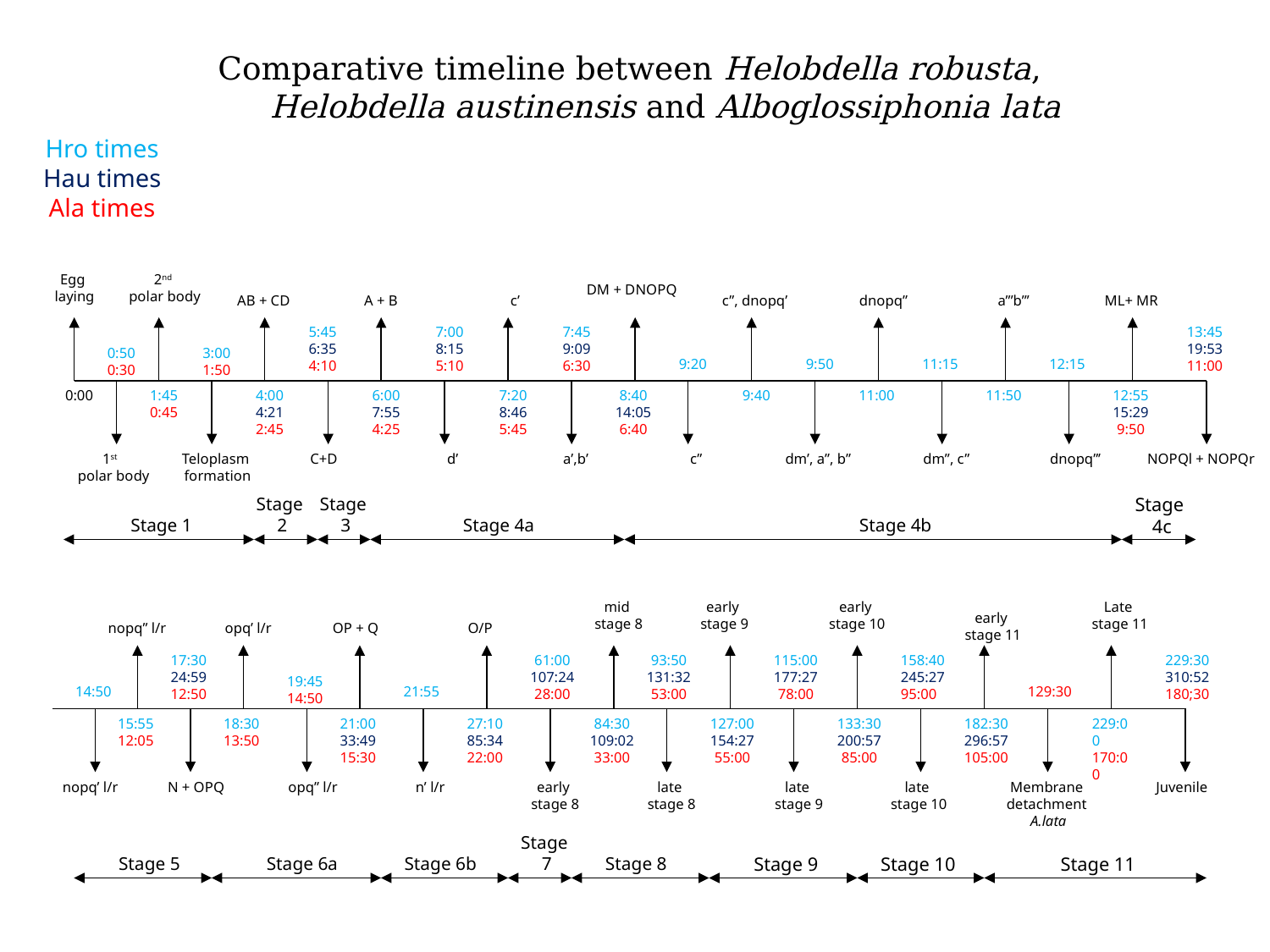

Comparative timeline between Helobdella robusta, Helobdella austinensis and Alboglossiphonia lata
Hro times
Hau times
Ala times
Egg
laying
2nd
polar body
DM + DNOPQ
AB + CD
A + B
c’
c”, dnopq’
dnopq”
a”’b”’
ML+ MR
5:45
6:35
4:10
7:00
8:15
5:10
7:45
9:09
6:30
13:45
19:53
11:00
0:50
0:30
3:00
1:50
9:20
9:50
11:15
12:15
0:00
1:45
0:45
4:00
4:21
2:45
6:00
7:55
4:25
7:20
8:46
5:45
8:40
14:05
6:40
9:40
11:00
11:50
12:55
15:29
9:50
1st
 polar body
Teloplasm
formation
C+D
d’
a’,b’
c”
dm’, a”, b”
dm”, c”
dnopq”’
NOPQl + NOPQr
Stage
2
Stage
3
Stage
4c
Stage 1
Stage 4a
Stage 4b
mid
stage 8
early
stage 9
early
stage 10
Late
stage 11
early
stage 11
nopq” l/r
opq’ l/r
OP + Q
O/P
17:30
24:59
12:50
61:00
107:24
28:00
93:50
131:32
53:00
115:00
177:27
78:00
158:40
245:27
95:00
229:30
310:52
180;30
19:45
14:50
14:50
21:55
129:30
15:55
12:05
18:30
13:50
21:00
33:49
15:30
27:10
85:34
22:00
84:30
109:02
33:00
127:00
154:27
55:00
133:30
200:57
85:00
182:30
296:57
105:00
229:00
170:00
nopq’ l/r
N + OPQ
opq” l/r
n’ l/r
early
stage 8
late
stage 8
late
stage 9
late
stage 10
Membrane
detachment
A.lata
Juvenile
Stage
7
Stage 5
Stage 6a
Stage 6b
Stage 8
Stage 9
Stage 10
Stage 11
